# Spiking neural network with working memory can integrate and rectify spatiotemporal features

**DOI:** 10.3389/fnins.2023.1167134

**Published:** 2023-06-14

**Authors:** Yi Chen, Hanwen Liu, Kexin Shi, Malu Zhang, Hong Qu

**Affiliations:** School of Computer Science and Engineering, University of Electronic Science and Technology of China, Chengdu, China

**Keywords:** spiking neural network, working memory, convolutional neural network, CIFAR10, multi-dendrite

## Abstract

In the real world, information is often correlated with each other in the time domain. Whether it can effectively make a decision according to the global information is the key indicator of information processing ability. Due to the discrete characteristics of spike trains and unique temporal dynamics, spiking neural networks (SNNs) show great potential in applications in ultra-low-power platforms and various temporal-related real-life tasks. However, the current SNNs can only focus on the information a short time before the current moment, its sensitivity in the time domain is limited. This problem affects the processing ability of SNN in different kinds of data, including static data and time-variant data, and reduces the application scenarios and scalability of SNN. In this work, we analyze the impact of such information loss and then integrate SNN with working memory inspired by recent neuroscience research. Specifically, we propose Spiking Neural Networks with Working Memory (SNNWM) to handle input spike trains segment by segment. On the one hand, this model can effectively increase SNN's ability to obtain global information. On the other hand, it can effectively reduce the information redundancy between adjacent time steps. Then, we provide simple methods to implement the proposed network architecture from the perspectives of biological plausibility and neuromorphic hardware friendly. Finally, we test the proposed method on static and sequential data sets, and the experimental results show that the proposed model can better process the whole spike train, and achieve state-of-the-art results in short time steps. This work investigates the contribution of introducing biologically inspired mechanisms, e.g., working memory, and multiple delayed synapses to SNNs, and provides a new perspective to design future SNNs.

## 1. Introduction

**Figure 1:**
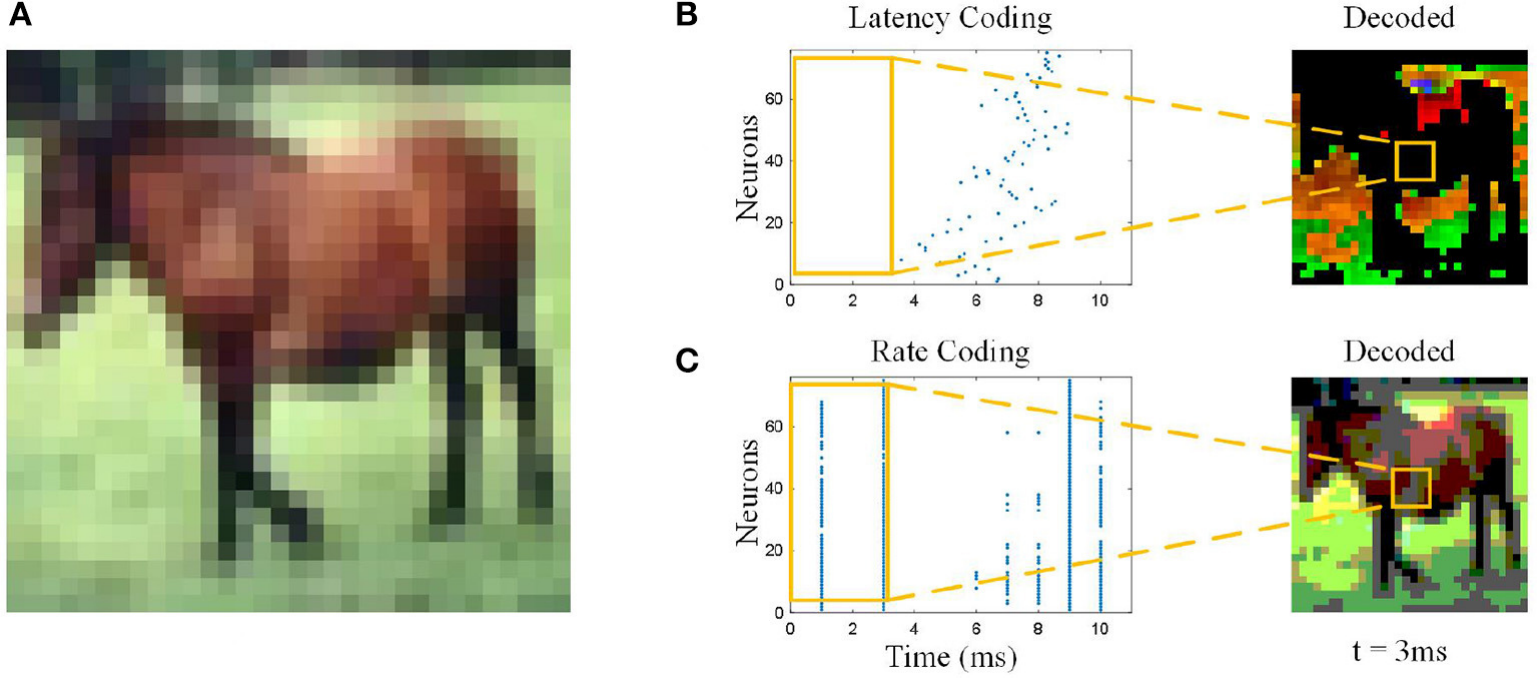
Information loss with different coding methods. **(A)** Original image. The pixel value of the image will be directly input into ANNs, so the ANNs see the full image. **(B)** Latency coding. At 3 ms, the neurons “see” only part of the background grass in the picture, not the horse, and the color was distorted. **(C)** Rate coding. At 3 ms, the neurons “see” the shape of the horse, but the color was distorted.

Artificial Neural Networks (ANNs) learned from biological neural networks achieved huge success in these years. Spiking Neural Networks (SNNs) as a step forward to biological neural networks caught up with their ANNs counterparts and even outperform in computer vision (Meng et al., 2022; Zhu et al., 2022), sound recognition (Pan et al., 2020, 2021), and so on (Lobo et al., 2020; Li et al., 2022). Theoretically, SNNs, which are more similar to biological neural networks, should have more advantages in dealing with real tasks. On the contrary, there is still a certain gap between SNN and ANN in terms of the scope of application and performance in general. The reason is that ANN's synchronicity in processing simulated information allows it to fully consider every detail. In contrast, SNN's asynchronous processing of discrete information makes it better in power consumption performance, but it cannot comprehensively consider the complete information. In fact, SNNs' advantages in complex temporal dependence have not been fully discovered. A classic spiking neuron can only accumulate the most recent spike train it has received and fails to integrate comprehensive spatiotemporal features, as shown in Figure 1. For the static image with rate-based coding, the one-way aggregation of SNN itself makes it unable to judge based on valid information. The effect of this problem is even more pronounced with latency coding, where neurons see only a small part of the picture. The same is true for dynamic sequential data. For example, if a video of a long jump contains two consecutive actions, namely a run-up, and a jump, the traditional SNN structure may make a judgment during the run-up and ignore the subsequent jump.

Previously, researchers have tried to increase the temporal receiver domain of SNNs in various ways to improve the ability of SNNs to process spatiotemporal data. According to the different ideas of their methods, they can be divided into two categories: training more parameters of neurons and changing the structure of neural networks.

In addition to the weights that can be trained as in ANN, spiking neurons have many different parameters that determine the dynamic characteristics of neurons. In Luo et al. (2022), by training synaptic delay, the neuron obtains the ability to rearrange and integrate the spike train, which further increases the ability of SNN to process timing data based on the original learning algorithm. In Fang et al. (2021b), by training the time constant of the neuron, the spike response function of the neuron is changed, and the spiking neuron can obtain the time receiving field length and attenuation coefficient more suitable for the current task through learning. In Rathi and Roy (2023), the threshold is changed into a trainable parameter. However, the above algorithm is only optimized at the neuron level, and a single neuron can still only obtain limited information and cannot obtain a global perspective.

In order to expand spiking neurons' temporal receptive fields, researchers have made various works according to specific tasks by referring to various ANN structures that already exist. In Zhang and Li (2021), the author added circular connections to SNN, but the weight of phantom connections was manually set. Zhang and Li (2019), the author also changed the network into a loop structure and proposed an effective training method. El-Assal et al. (2022), the author takes the 3D convolution kernel as the input weight. However, this method will extract a large amount of redundant information between adjacent time periods, so compared with the 2D convolution method commonly used in SNN, the improvement is limited. In Yao et al. (2021), the author introduced the attention structure into SNN and proposed the SNN network based on temporal attention. This kind of network is a hybrid network of ANN and SNN. In the forward inference stage, in addition to the original SNN operation, the network also needs to pass a fully connected network with a multi-layer sigmoid function as the activation function, which increases the computation amount.

In the biological brain, cortical neurons process information on multiple timescales, and areas important for working memory contain neurons capable of integrating information over a long timescale (Kim and Sejnowski, 2021). In terms of vision, working memory is already involved in the early part of the whole visual pathway. The distinct visual stimuli (oriented gratings and moving dots) are flexibly recorded into the same working memory format in visual and parietal cortices when that representation is useful for memory-guided behavior (Kwak and Curtis, 2022). Therefore, working memory is very important for the extraction of temporal information. Introducing working memory into SNN can effectively improve the processing ability of SNN on spatiotemporal data. Although the specific structure of working memory in the brain has not been determined, we can still combine the properties of SNN to propose a working memory block suitable for SNN.

Based on this, we integrate multiple delayed synapses in spiking neural networks and propose a simple but effective structure Spiking Neural Network with Working Memory (SNNWM). Compared with traditional SNN, SNNWM adds multiple groups of dendrites with different delays. These dendrites effectively increase SNN's receptive field in the time domain, enabling SNN to gradually acquire global vision with the deepening of network layers. After that, we provide a simple method to implement the proposed network architecture in both software and hardware. Among them, we analyze the differences between SNNWM and traditional SNN in hardware implementation and draw the conclusion that SNNWM can increase a small number of storage resource access operations without increasing the extra consumption in computation. Finally, we test our method on two different data: static image, and dynamic event sequence. Experimental results show that working memory increases SNN's power dealing with spike trains and reaches the state of the art with low latency.

In summary, our main contributions are as follows:

1) We propose the spiking neural network with working memory by introducing multiple delayed synapses and offer a simple method to reduce the number of parameter increases.
2) For the model proposed in this paper, we give the implementation methods of software and hardware and further demonstrate that the proposed model will not generate excessive resource consumption when implemented by hardware.
3) We further validated the effectiveness of adding working memory to SNN by testing the effectiveness of the proposed model on static and dynamic data, respectively.

## 2. Materials and methods

In this section, we first introduce the spiking neuron model and later propose our spiking neural network with working memory based on this neuron model. After that, we propose a practical implementation of SNNWM for neuromorphic hardware as well as FPGAs (Field Programmable Gate Arrays). Subsequently, to further enhance the temporal aggregation ability of the model and simplify the computational burden, we propose a temporal fusion layer. Finally, we introduce the training algorithm used in this paper.

### 2.1. Spiking neuron models

The Leaky Integrate and Fire (LIF) model and Integrate and Fire (I&F) model are the two most commonly used spiking neuron model at present, which is more optimized for neuromorphic hardware design due to their lower complexity and iterative representation. In general, the I&F model can be regarded as the LIF model with the leaky term set to 1. Therefore, for simplicity and generality, we adopt the discrete representation LIF model in Wu et al. (2018). In LIF model, the membrane potential *V*_*j*_ of neuron *j* at time *t* is updated as follow:


(1) $$\begin{array}{l} V_{j}\left(t\right)=e^{-\frac{1}{\tau }}V_{j}\left(t-1\right)+\overset{N_{i}}{\underset{i=1}{\sum }}w_{ij}K\left(s_{i}\left(t\right)\right), \end{array}$$

where *w*_*ij*_ is the synaptic weight between neuron *i* and *j*, *K*(*s*) is the spike response function and here we chose *K*(*s*) = *s* for simplicity, *s*_*i*_(*t*) ∈ {0, 1} is the spike train from presynaptic neuron *i* and *s*(*t*) = 1 means neuron *i* fires a spike at time *t*. When the membrane potential exceeds the threshold θ from below, the spiking neuron *j* fires a spike *s*_*j*_(*t*) at this time *t*, and its membrane potential is set to resting potential *V*_*rest*_. This procedure can be described as:


(2) $$\begin{array}{l} s_{j}\left(t\right)=H\left(V_{j}\left(t\right)-\theta \right), \end{array}$$

(3) $$\begin{array}{l} V_{j}\left(t\right)=V_{j}\left(t\right)\left(1-s_{j}\left(t\right)\right)+V_{rest}s_{j}\left(t\right), \end{array}$$

where *H* is the Heaviside step function:


(4) $$H(x)=\{\begin{array}{ll} 1, & x\geq 0\\ 0, & else \end{array}.$$

**Figure 2:**
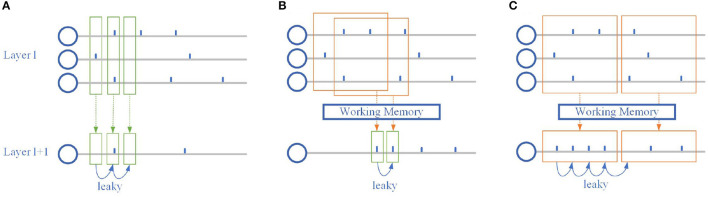
Temporal dependence of the spiking neuron. **(A)** The classic spiking neuron model. The output spike train is directly affected by the current input and indirectly by the previous input. **(B)** Spiking neuron with overlapping working memory. The output spike train is directly affected by both the current and the previous input. **(C)** Spiking neuron with individual working memory. The output spike train in the same working memory window is rectified.

It can be found by Equation (1) that SNN can process simple time-series information naturally, and its temporal reception field is closely related to constant τ, namely leaky term. Specifically, the previous spikes can affect the membrane potential at that moment, dotted arrows from layer *l* to layer *l*+1 in Figure 2A, and then indirectly affect the membrane potential at the current moment by leaky term, the solid arrows on the bottom in Figure 2A. Moreover, this indirect effect may be eliminated by firing a spike that causes the membrane potential to reset. Spiking neurons themselves have limited temporal information processing ability, so it is necessary to make some changes in the network structure to improve the ability of SNN.

### 2.2. Spiking neural network with working memory

Working memory is defined as a processing resource of limited capacity, involved in the preservation of information while simultaneously processing the same or other information. There are a variety of theories about the formation mechanism and storage structure of working memory. Still, here we only focus on its main function, which is to store a piece of related information for other modules to use. Here, we expect the spiking neuron to change its membrane potential mainly based on the spikes over a period of time, rather than on the spikes at the present moment. This means that we need to modify the spike train over a period of time so that it can reach the neuron at the same time.

In the biological brain, there are multiple synapses between neurons, and these diverse synapses increase the brain's ability to process complex spatial-temporal signals. In ANNs, there is only one synaptic connection between two neurons to simplify the model and facilitate calculation. Even if there are multiple synapses, because of the way ANN works synchronously, multiple synapses can be equivalent to one synaptic connection. On the contrary, in SNN, the spiking neurons' temporal dynamics enable multiple delayed synapses effectively increasing the ability of SNN to process complex spatial-temporal data.

Inspired by multiple delayed synapses in Bohte et al. (2000), we integrate multiple groups of dendrites with different delays based on the original LIF model and proposed a multi-dendrite spiking neural network with delay. Equation (1) becomes:


(5) $$\begin{array}{l} V_{j}\left(t\right)=e^{-\frac{1}{\tau }}V_{j}\left(t-1\right)+\overset{N_{k}}{\underset{k=1}{\sum }}\overset{N_{i}}{\underset{i=1}{\sum }}w_{ijk}s_{i}\left(t-d_{k}\right), \end{array}$$

where *w*_*ijk*_ and *d*_*k*_ is the weight and transmission delay of dendrite group *k*, respectively. Multiple groups of dendrites with different delays effectively increase the time domain exploration of the neuron's input spikes at the same synapse. As shown in Figures 2B, C, Multiple dendrites help spiking neurons explore many different combinations of spike trains simultaneously, without having to wait for all inputs to proceed to the next layer of computation as in ANN-SNN hybrid networks that introduce the attention.

This multi-dendrite structure would increase the number of SNNs' parameters and further reduce usage in resource-restricted edging platforms. Inspired by spatial factorization in Inception-V2 (Szegedy et al., 2016), we split the weight matrix *w*_*ijk*_ of size *N*_*i*_ × *N*_*j*_ × *N*_*k*_ in Equation (5) into two matrices of size *N*_*i*_ × *N*_*j*_ × 1 and 1 × *N*_*k*_, respectively. In this way, the number of network parameters is reduced from the original *N*_*i*_ × *N*_*j*_ × *N*_*k*_ to *N*_*i*_ × *N*_*j*_ + *N*_*k*_ and only increased by *N*_*k*_ compared with classical SNN. In this way, Equation (5) becomes:


(6) $$\begin{array}{l} V_{j}\left(t\right)=e^{-\frac{1}{\tau }}V_{j}\left(t-1\right)+\overset{N_{k}}{\underset{k=1}{\sum }}w_{k}\overset{N_{i}}{\underset{i=1}{\sum }}w_{ij}s_{i}\left(t-d_{k}\right), \end{array}$$

To further enhance the global processing capability of SNN, we introduce an additional memory mechanism to rectify spike train through working memory to increase synchronization, as shown in Figure 2C. The rectified output spike train can effectively increase the stability of the neural network and improve the ability to handle static data, and Equation (6) becomes:


(7) $$\begin{array}{l} V_{j}\left(t\right)=e^{-\frac{1}{\tau }}V_{j}\left(t-1\right)+\overset{N_{k}}{\underset{k=1}{\sum }}w_{k}\overset{N_{i}}{\underset{i=1}{\sum }}w_{ij}s_{i}\left(t-d_{k}-m\right), \end{array}$$

(8) $$\begin{array}{l} m=\left\lfloor t/mem\text{\_}len\right\rfloor , \end{array}$$

where *mem*_*len* is the length of working memory. The working memory that operates in this way takes into account all the spikes within *mem*_*len* and continuously feeds them into the spiking neuron for *mem*_*len*. This method can effectively alleviate the information loss caused by uneven spike distribution in the time domain for data that do not need fast time-varying information.

### 2.3. Temporal fusion layer

**Figure 3:**
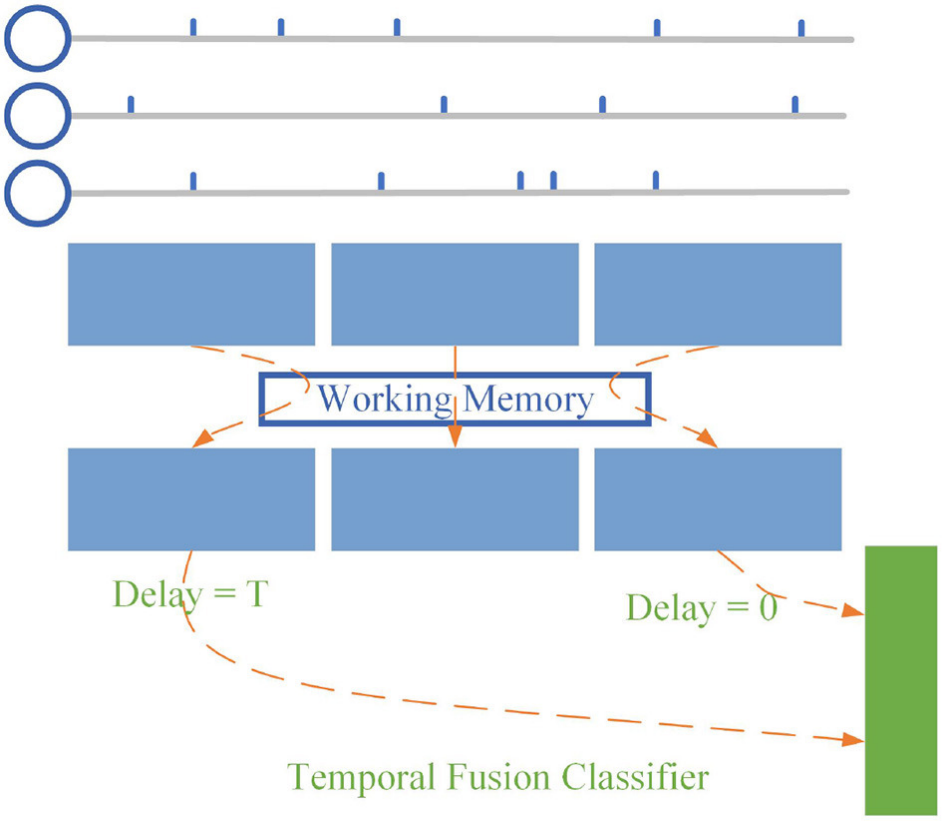
Temporal fusion layer. Instead of using the dynamic processes inside the SNN, the temporal fusion layer combines pulses at all times to give the SNN a global view.

At present, most SNNs take the spike frequency or average membrane potential of the last layer as output when processing tasks. We designed the final layer based on the proposed SNNWM. For spike sequences with fixed input length *T*, we set the dendrite groups' transmission delay of neurons at the last layer as {0, 1, 2..., *T*}, as shown in Figure 3 and only the mode potential of the *T*th time step is used as the output. According to Equation (4), the decoding scheme can be expressed as:


(9) $$\begin{array}{l} o_{j}=\overset{T}{\underset{k=1}{\sum }}w_{k}\overset{N_{i}}{\underset{i=1}{\sum }}w_{ij}s_{i}\left(T-d_{k}\right). \end{array}$$

In this way, on the one hand, the neurons in the last layer obtain spike information at all times at the time step *T*, thus enhancing the performance of SNN; on the other hand, the computation at the previous *T*−1 time step is reduced, effectively reducing the computation cost.

### 2.4. Implementation in software and hardware

Our goal is to make the most of the SNN's low-power, high-dynamic processing capabilities, without having to be the same as biological neurons. Therefore, in the process of practical application, the {0, 1} sequence is often used to encode the spike sequence. A 0 or 1 in each bit represents whether there is a spike event, and each position represents a small period. For example, a spike train 010110 can represent a spike train with a simulation duration of 6 ms and each period of 1 ms.

To further simplify the model, we use an arithmetic sequence as the delay in SNNWM. That is, the synaptic delay between the two neurons is {0, 1, 2, 3...*L*}, where L represents the length of working memory. And the synaptic delay between the two adjacent layers of neurons was the same. In this way, SNNWM with overlapping working memories can be achieved by a simple one-dimensional convolution operation with an additional convolution kernel *W*1, or *w*_*k*_ in Equation (6), in the time dimension.

The implementation method of SNNWM without overlap is shown in the pseudo-code of Algorithm 1. Firstly, the input spike train *S*_*in*_ is divided into *N* segments of length *L* in the time dimension. These segments can be processed in parallel with each other before calculating changes in membrane potential *V*. The change in membrane potential *I*_*seg*_ from the input is obtained by multiplying the spike segments with the synaptic weights of sizes *N*_*i*_ × *N*_*j*_ and *N*_*k*_, respectively. The change in membrane potential from the input is obtained by multiplying the spike segment with the synaptic weight *W*1 of size *N*_*i*_ × *N*_*j*_ × 1 and the synaptic weight *W*2 of size 1 × *N*_*k*_.

**Algorithm 1:**
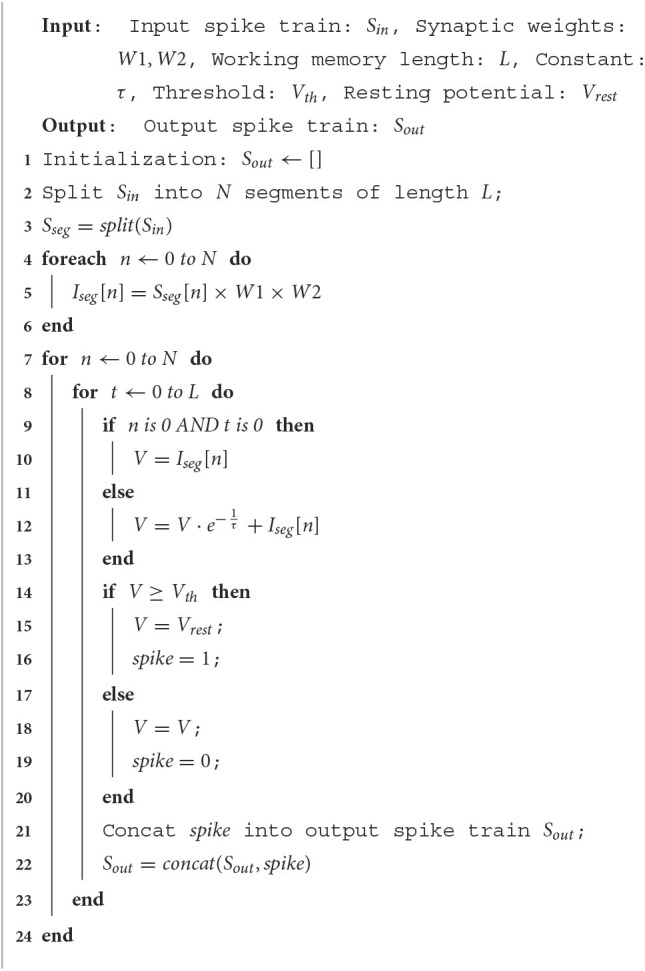
Pseudo code of SNNWM.

**Figure 4:**
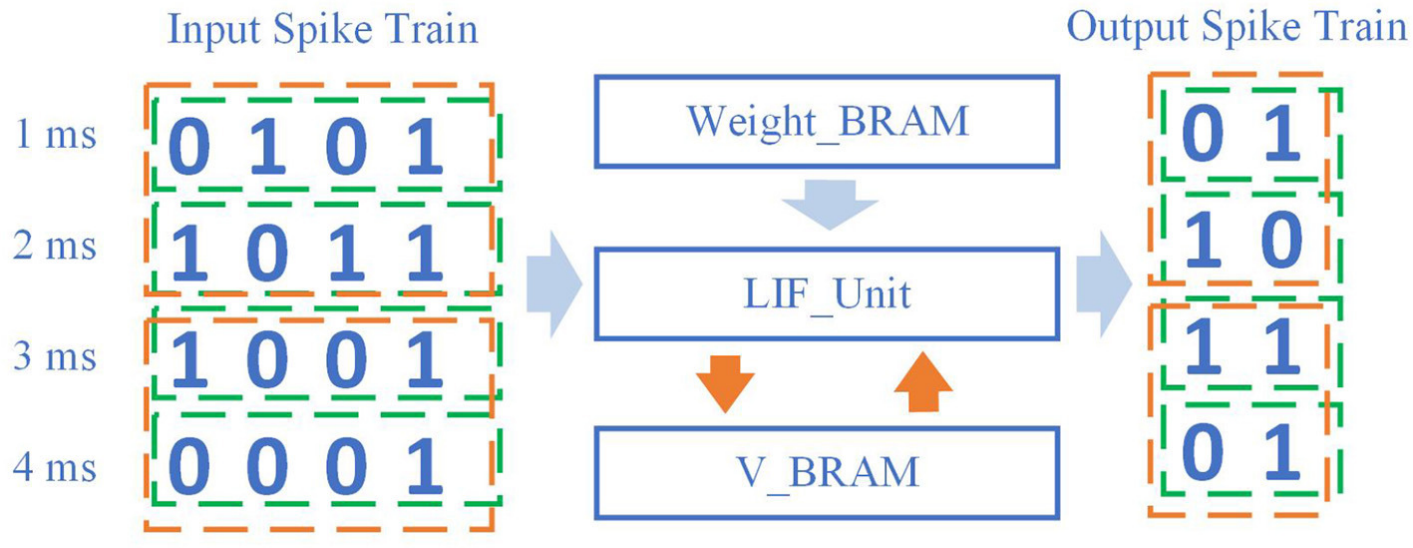
Implementation of working memory in hardware. Compared with classical methods, this method does not consume too much in global data transmission and processing. The computation cost by the input spike train and the change of neuron state remains unchanged.

The method of SNNWM's hardware implementation, specifically FPGA, is shown in Figure 4. The traditional SNN will input the spike train at each moment (the sequence in the green box) into the LIF unit, and extract the weight matrix and the membrane potential at the last moment from BRAM. After completing the calculation of the membrane potential at the current moment, the obtained spike train at the current moment will be output, and then the new neuron membrane potential will be stored in V_BRAM.

The total input spike data size remains the same, and the output spike train data size remains the same. It is worth noting that since the introduction of working memory will simultaneously participate in the calculation of spikes within each working memory window size, the fetch operation of V_BRAM only occurs at the beginning of this window time, and the save operation only occurs at the end of this window time. Compared with the previous SNN, the number of V_BRAM accesses is saved.

### 2.5. Training

For the optimization of network parameters, we choose the STBP (Spatio-Temporal BackPropagation) (Wu et al., 2018) in the surrogate gradient learning method. At present, there are many opinions about the selection of approximate functions. Wu et al. (2018) believes that the parameters of the function are more important than the selection of the function, while Fang et al. (2021a) believes that the approximate function with different shapes can bring better results. In this work, we chose the surrogate function:


(10) $$\begin{array}{l} \frac{\partial H\left(x\right)}{\partial x}=e^{-2x^{2}} \end{array}$$

for gradient learning. Compared with the triangular or rectangular surrogate function, the exponential surrogate function can ensure that some gradient information can be transmitted even when the membrane potential is far from the threshold, rather than no gradient at all. At the same time, we found in the experiment that using hard Tan as an approximation function would slightly increase the training time and have no effect on the final result.

## 3. Results

To verify the ability of our proposed model to extract temporal and spatial features, we designed a variety of different experiments on two different data types: images and event streams. We compared the proposed method with other different methods with the same or similar network structure and scale. These networks include conventional network structures or are optimized according to the characteristics of SNNs, introducing particular neurons or layer structures. The training methods adopted by them include transform-based methods and BP-based methods. The details of the implemented methodology and the experimental setup are presented below. After that, we carried out experiments for different types of data sets and comprehensively tested the influence of preprocessing methods and network working memory size on the model's performance.

### 3.1. Implementation details

In the following experiments, we implemented the proposed model on two NVIDIA RTX 3090s using the Pytorch training framework. For CIFAR10 (Krizhevsky et al., 2017) and CIFAR100 (Krizhevsky et al., 2017), we utilize the SGD optimizer with the momentum of 1*e*^−4^ to accelerate the training process, and for DVS128 Gesture (Amir et al., 2017) and CIFAR10-DVS (Li et al., 2017), we utilize the Adam optimizer. The hyperparameters used for training are shown in Table 1 for different data sets. Compared with some other SNN-related works, we adopt the conventional training parameter setting here, and all experimental results are the average values obtained after 5 repetitions with different random seeds.

**Table 1:** Hyper parameters.

**Hyper parameter**	**CIFAR10**	**CIFAR100**	**DVS128 Gesture**	**CIFAR10-DVS**
Training epoch	200	300	300	300
Batch size	128	128	16	32
Learning rate	1*e*^−1^	1*e*^−1^	1*e*^−3^	1*e*^−3^
Time steps	6	6	16	10

To ensure the fairness of the comparison, we choose different network structures for different tasks to test, as shown in Table 2. In this table, C represents the convolutional layer, MP represents the max pooling layer, AP represents the average pooling layer, GAP represents the global average pooling layer, and pure numbers represent the fully connected layer. The number before all symbols represents the number of output channels or neurons, and the number after symbols represents the size of the kernel. Among them, for the task of image class, spatial information is more important than temporal information, and we adopt the residual structure of Fang et al. (2021a). In the training process of the SNN network, compared with the layer-by-layer stacked VGG structure, the gradient information of the residual structure can be transmitted through shortcuts, which can effectively alleviate the gradient error caused by the surrogate gradient function. For the event stream data, time characteristics and spatial characteristics are equally important, we use the VGG structure network to avoid the time characteristics in the event stream being disrupted.

**Table 2:** Network structures.

**Dataset**	**Architecture**	**Detail**
CIFAR10	SEW ResNet18	64C3-MP2-64SEWblock*2-128SEWblock*2 - 256SEWblock*2-512SEWblock*2-GAP-10
CIFAR100	SEW ResNet18	64C3-MP2-64SEWblock*2-128SEWblock*2 - 256SEWblock*2-512SEWblock*2-GAP-100
DVS128 gesture	VGG-small	128C3-MP2-128C3-MP2-128C3-MP2- 128C3-MP2-512-11
DVSCIFAR10	VGG	16C3-AP2-32C3-AP2-64C3-64C3-AP2-128C3- 128C3-AP2-128C3-128C3-AP2-256-10

### 3.2. Static data

As previously analyzed, SNNs are limited in their ability to process even static data if they cannot make valid judgments based on the entire spike train. Here, we select the benchmark of two static image classification tasks: CIFAR10 and CIFAR100 to verify that the proposed model is simple and effective. For the still image data, we did not adopt additional data augmentation methods for pulse sequences according to the time-dependent characteristics of SNN and only used data augmentation methods for the original image data. Specifically, we use Autoaugment (Cubuk et al., 2019) as an augmentation to improve the accuracy of image classification models. Compared to the classical crop adopted in most previous works, With random horizontal flipping and normalization, using auto augmentation improves the final classification result on CIFAR10 by about 0.4%.

#### 3.2.1. Comparison with prior works

For image-type data, the main factor affecting the classification results is the spatial feature extraction ability of the model. Therefore, most of the work in this area focuses on ensuring the accuracy of the information represented by the spike train during the forward propagation or the accuracy of the gradient information in the backward propagation. As shown in Table 3, compared with other methods, our method only needs 6 timesteps to achieve the classification accuracy of 95.41% on CIFAR10 and 78.77% on CIFAR100.

**Table 3:** Classification accuracy on static data.

**Dataset**	**Proposals**	**Architecture**	**Timesteps**	**Accuracy(%)**
CIFAR10	Rathi and Roy, 2023	VGG16	10	93.44
	Rathi and Roy, 2023	ResNet20	10	92.54
	Wu J. et al., 2021	CifarNet	8	90.98
	Fang et al., 2021a	VGG	8	93.50
	Zheng et al., 2021	ResNet19	6	93.16
	Deng et al., 2022	ResNet19	256	94.50
	This work	SEW-ResNet18	6	95.41
CIFAR100	Rathi et al., 2020	VGG11	125	67.87
	Rathi and Roy, 2023	ResNet20	5	64.09
	Deng et al., 2022	ResNet19	6	74.72
	This work	SEW-ResNet18	6	78.77

#### 3.2.2. Effects of different encoding methods

**Figure 5:**
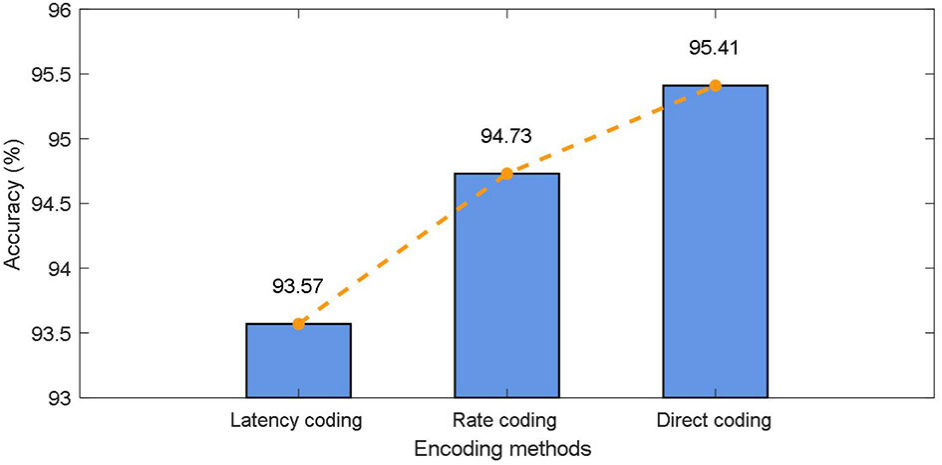
Effects of encoding methods in CIFAR10.

The commonly used static data coding methods include direct coding, rate coding, and latency coding. To be specific: (1) Direct coding is to input the pre-processed data directly into SNN as the current at every moment. (2) The commonly used method in rate coding is to encode the preprocessed data into the pulse train of Poisson distribution, where the expectation of Poisson distribution is related to the data value. (3) Latency coding is to map the pre-processed data size directly to the specific pulse firing moment, in which case the neuron fires only once. As mentioned before in this paper, different coding schemes have a significant impact on the classification results of the model in the classical SNN model, here, we test the effect of different coding methods on the CIFAR10 dataset as shown in Figure 5.

It can be found that, on the one hand, the direct encoding approach achieves the best results. The direct encoding scheme is more biologically interpretable. The photoreceptor neurons used to convert light signals into spike trains already have preliminary feature extraction capabilities in biological retinas. On the other hand, the proposed model achieves better classification results with different coding schemes. In particular, when latency coding is used, there is less drop in accuracy relative to direct encoding. This is attributed to the fact that the working memory in the proposed method integrates the input information at multiple time steps, allowing the model to obtain a larger field of view.

#### 3.2.3. Effects of working memory length

**Figure 6:**
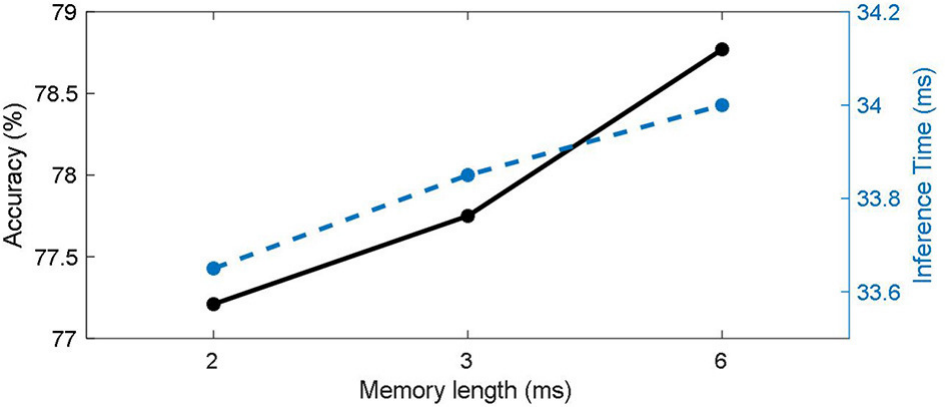
Effects of working memory length in CIFAR100.

The length of the working memory will affect the number of parameters that the model has, as well as its ability to integrate temporal information. We tested different working memory lengths separately on CIFAR100 with direct coding method, and the results are shown in Figure 6. It can be seen that the classification accuracy (solid black line) increases with memory length. For static data, longer working memory can give the model more time integration ability, which is also beneficial to the stability of the model. Therefore, the best classification results were obtained for networks with memory lengths up to 6 timesteps of the simulation duration. On the other hand, the reasoning time for a single batch (blue dashed line) increases with memory length.

### 3.3. Sequence data

Furthermore, we verify the effect of the proposed model on sequential data where time correlation is more important. Here, we choose two datasets, DVS128 Gesture and CIFAR10-DVS, for testing. In the CIFAR10-DVS dataset, the information of the original picture in the CIFAR10 dataset is obtained through motion, and the short-term information is more critical.

#### 3.3.1. DVS data encoding

A variety of coding schemes exist for event streams, including time-surface (Lagorce et al., 2017), timestamp (Huang, 2021), and so on (Sabater et al., 2022; Wang et al., 2022). Here we use the more common approach in SNNs. Specifically, we use the encoding method in SpikingJelly (Fang et al., 2020) to convert the event stream data into multiple consecutive pictures. After that, the direct input coding method was used, that is, the image pixels were normalized and directly fed into the SNN as the input current.

The data augmentation method's impact on the results of event stream data is important. In this paper, to reduce the training cost and save time, we convert the event stream data into image data and then use the data augmentation method. In this article, we will use a data augmentation approach similar to that commonly used for still images. Specifically, for the DVS128 Gesture dataset, we used a random crop. For the CIFAR10-DVS data set, random crop and random horizontal flipping were used.

#### 3.3.2. Comparison with prior works

For sequential event stream data, local and global temporal information is equally important. Local temporal information can be captured by using the time-varying property of spiking neurons, but the long-term information may disappear due to discrete neuron firing. Therefore, most works enhance the ability of SNN to process event streams by changing the neuron model or adding modules that can obtain long-term information, such as recurrent structures or attention modules.

It can be seen from Table 4 that our proposed method achieves a classification accuracy of 98.26% on the DVS128 Gesture data set and 80.1% on the CIFAR10-DVS data set.

**Table 4:** Classification accuracy on sequence data.

**Dataset**	**Proposals**	**Architecture**	**Timesteps**	**Accuracy (%)**
DVS128 gesture	Zheng et al., 2021	CifarNet	40	96.87
	Wu Z. et al., 2021	VGG	60	97.56
	Fang et al., 2021b	ResNet19	20	97.57
	Fang et al., 2021a	ResNet19	16	97.92
	This work	VGG-small	16	98.26
CIFAR10-DVS	Wu Z. et al., 2021	VGG	10	70.4
	Fang et al., 2021b	VGG	20	74.8
	Yao et al., 2021	VGG	10	72.0
	Fang et al., 2021a	Wide-7B-Net	16	74.4
	This work	VGG	10	80.1

#### 3.3.3. Effects of working memory length

**Figure 7:**
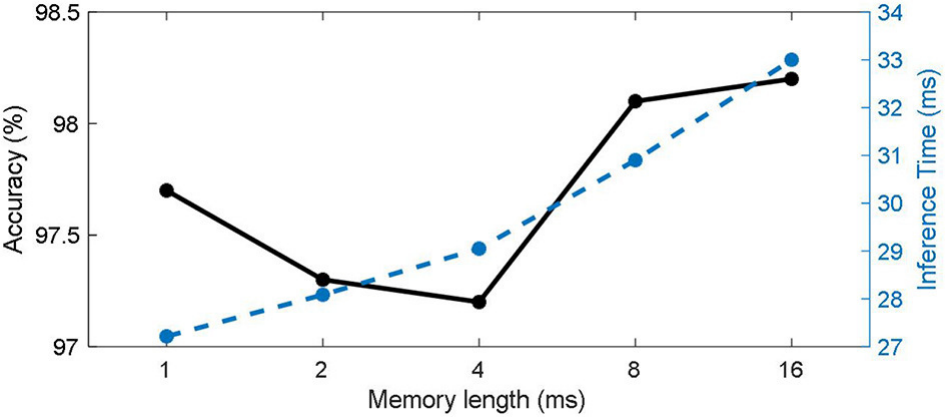
Effects of working memory length in DVS128 Gesture.

Event stream data is different from static images in that in addition to spatial features, temporal features are also always required, so the model's ability to extract temporal features has a significant impact on the final classification results. The working memory size affects the model's ability to extract temporal features. We tested it on the DVS128 Gesture dataset and the results are shown in Figure 7. It can be seen that with the increase in working memory size, the classification accuracy (solid black line) of the model shows a trend of decreasing now and then increasing. This is partly because the size of the working memory matches the length of key features of the data itself. When the working memory size is small, the model mainly classifies by spatial features. As working memory size grows, models tend to make judgments based on certain key short-term features, ignoring global information. As the working memory grows, the model acquires a global view and can classify actions based on the sequence. This is also in line with the example given at the beginning of the article. On the other hand, the inference time for a single batch (blue dashed line) increases with memory length.

## 4. Discussion

In this paper, we first analyze the key problems that affect SNN network processing spatiotemporal data, namely, SNN can only aggregate information in one direction, and the temporal receiver field is limited. To solve this problem, we introduce working memory into SNN, propose a new network structure by combining multi-delay synapses, and give an effective method to reduce the number of parameters. Then, we provide the implementation method of the proposed model in software and hardware. Finally, we test the performance of the proposed model on several static and dynamic datasets. Experimental results show that SNN with working memory can effectively aggregate and rectify spatiotemporal features, thus improving the ability of SNN to process spatiotemporal data. Next, we discuss the effects of different encoding schemes on SNNWM, the effects of working memory length on SNNWM's inference speed and storage, and potential improvements for future SNNWM.

First, for static data, we analyzed the effects of three different coding schemes on SNNWM and found that direct coding had the best effect, while delayed coding had the worst effect, which was consistent with the results of most SNN-related studies. For direct coding, SNNWM achieved the highest classification accuracy because the same full precision value is stably entered at every moment. For rate coding and latency coding, since the input is a binary spike train and the simulation length determines the precision of the data received by SNNWM, so there will be certain performance degradation. The performance gap between the two is mainly due to the fact that rate coding has more input spikes than latency coding and the randomness in coding. For example, the Poisson distribution-based method used in this paper is equivalent to an augmentation of the training data and reduces overfitting.

Secondly, the length of Memory has a certain influence on the SNNWM's inference speed and storage. The introduction of working memory adds additional data slice and matrix multiplication operations and the storage grows as memory length increases. In particular, the additional memory consumption caused by working memory is approximately the same as adding an additional matrix multiplication of the size related to memory length to the original spiking neuron. Therefore, there is a trade-off between performance and resource consumption. At the same time, it can be found in the experiment on sequence data that the influence of memory length on model performance is not linear, so it is important to choose the appropriate memory length according to specific tasks.

Finally, SNNWM, as a neuron-level model refinement, can be used in various SNN network structures, such as the latest transformer-based model. At the same time, the learning algorithm used in this paper is based on BPTT (Back-Propagation Through Time) method, and the gradient needs to be transmitted step by step in time. Since SNNWM can be regarded as processing the spike train segment by segment, it is possible to try the gradient feedback at a segment level to reduce the training time and cost. Besides, this work is only a preliminary attempt at the visual classification, which can be extended to other types of data, such as speech, natural language processing, automatic driving, etc.

## Data availability statement

The original contributions presented in the study are included in the article/supplementary material, further inquiries can be directed to the corresponding author.

## Author contributions

YC proposed the idea, performed the experiments, and wrote the manuscript. All authors contributed to the experiment's design, result interpretation, and writing. All authors contributed to the article and approved the submitted version.

## References

[B1] Amir A. Taba B. Berg D. J. Melano T. McKinstry J. L. di Nolfo C.. (2017). “A low power, fully event-based gesture recognition system,” in 2017 IEEE Conference on Computer Vision and Pattern Recognition (Honolulu, HI: IEEE Computer Society), 7388–7397. 10.1109/CVPR.2017.781

[B2] Bohte S. M. Kok J. N. La Poutré J. A. (2000). “Spikeprop: backpropagation for networks of spiking neurons,” in ESANN (Bruges), 419–424.

[B3] Cubuk E. D. Zph B. Mane D. Vasudevan V. Le Q. V. (2019). “Auto augment: Learning augmentation strategies from data,” in IEEE Conference on Computer Vision and Pattern Recognition (Long Beach, CA: Computer Vision Foundation; IEEE), 113–123. 10.1109/CVPR.2019.00020

[B4] Deng S. Li Y. Zhang S. Gu S. (2022). “Temporal efficient training of spiking neural network via gradient re-weighting,” in The Tenth International Conference on Learning Representations (OpenReview.net).

[B5] el Assal M. Tirilly P. Bilasco I. M. (2022). “2D versus 3D convolutional spiking neural networks trained with unsupervised STDP for human action recognition,” in International Joint Conference on Neural Networks (Padua: IEEE), 1–8. 10.1109/IJCNN55064.2022.9892063

[B6] Fang W. Chen Y. Ding J. Chen D. Yu Z. Zhou H.. (2020). Spikingjelly. Available online at: https://github.com/fangwei123456/spikingjelly (accessed February 02, 2023).

[B7] Fang W. Yu Z. Chen Y. Huang T. Masquelier T. Tian Y. (2021a). “Deep residual learning in spiking neural networks,” in Advances in Neural Information Processing Systems 34: Annual Conference on Neural Information Processing Systems 2021, eds M. Ranzato, A. Beygelzimer, Y. N. Dauphin, P. Liang, and J. W. Vaughan. p. 21056-21069. Available online at: https://proceedings.neurips.cc/paper/2021/hash/afe434653a898da20044041262b3ac74-Abstract.html

[B8] Fang W. Yu Z. Chen Y. Masquelier T. Huang T. Tian Y. (2021b). “Incorporating learnable membrane time constant to enhance learning of spiking neural networks,” in 2021 IEEE/CVF International Conference on Computer Vision (Montreal, QC: IEEE), 2641–2651. 10.1109/ICCV48922.2021.00266

[B9] Huang C. (2021). “Event-based timestamp image encoding network for human action recognition and anticipation,” in International Joint Conference on Neural Networks (Shenzhen: IEEE), 1–9. 10.1109/IJCNN52387.2021.9534386

[B10] Kim R. Sejnowski T. J. (2021). Strong inhibitory signaling underlies stable temporal dynamics and working memory in spiking neural networks. Nat. Neurosci. 24, 129–139. 10.1038/s41593-020-00753-w 33288909

[B11] Krizhevsky A. Sutskever I. Hinton G. E. (2017). ImageNet Classification with Deep Convolutional Neural Networks. New York, NY: Association for Computing Machinery. 10.1145/3065386

[B12] Kwak Y. Curtis C. E. (2022). Unveiling the abstract format of mnemonic representations. Neuron 110, 1822.e5–1828.e5. 10.1016/j.neuron.2022.03.016 35395195 PMC9167733

[B13] Lagorce X. Orchard G. Galluppi F. Shi B. E. Benosman R. B. (2017). HOTS: a hierarchy of event-based time-surfaces for pattern recognition. IEEE Trans. Pattern Anal. Mach. Intell. 39, 1346–1359. 10.1109/TPAMI.2016.2574707 27411216

[B14] Li H. Liu H. Ji X. Li G. Shi L. (2017). CIFAR10-DVS: an event-stream dataset for object classification. Front. Neurosci. 11, 309. 10.3389/fnins.2017.00309 28611582 PMC5447775

[B15] Li J. Li D. Jiang R. Xiao R. Tang H. Tan K. C. (2022). Vision-action semantic associative learning based on spiking neural networks for cognitive robot. IEEE Comput. Intell. Mag. 17, 27–38. 10.1109/MCI.2022.3199623

[B16] Lobo J. L. Del Ser J. Bifet A. Kasabov N. (2020). Spiking neural networks and online learning: an overview and perspectives. Neural Netw. 121, 88–100. 10.1016/j.neunet.2019.09.004 31536902

[B17] Luo X. Qu H. Wang Y. Yi Z. Zhang J. Zhang M. (2022). Supervised learning in multilayer spiking neural networks with spike temporal error backpropagation. IEEE Trans. Neur. Netw. Learn. Syst. 1–13. 10.1109/TNNLS.2022.3164930 35436200

[B18] Meng Q. Xiao M. Yan S. Wang Y. Lin Z. Luo Z. Q. (2022). “Training high-performance low-latency spiking neural networks by differentiation on spike representation,” in IEEE/CVF Conference on Computer Vision and Pattern Recognition (New Orleans, LA: IEEE), 12434–12443. 10.1109/CVPR52688.2022.01212

[B19] Pan Z. Chua Y. Wu J. Zhang M. Li H. Ambikairajah E. (2020). An efficient and perceptually motivated auditory neural encoding and decoding algorithm for spiking neural networks. Front. Neurosci. 13, 1420. 10.3389/fnins.2019.01420 32038132 PMC6987407

[B20] Pan Z. Zhang M. Wu J. Wang J. Li H. (2021). Multi-tone phase coding of interaural time difference for sound source localization with spiking neural networks. IEEE/ACM Trans. Audio Speech Lang. Process. 29, 2656–2670. 10.1109/TASLP.2021.3100684

[B21] Rathi N. Roy K. (2023). DIET-SNN: A low-latency spiking neural network with direct input encoding and leakage and threshold optimization. IEEE Trans. Neur. Netw. Learn. Syst. 34, 3174–3182. 10.1109/TNNLS.2021.3111897 34596559

[B22] Rathi N. Srinivasan G. Panda P. Roy K. (2020). “Enabling deep spiking neural networks with hybrid conversion and spike timing dependent backpropagation,” in 8th International Conference on Learning Representations (OpenReview.net).

[B23] Sabater A. Montesano L. Murillo A. C. (2022). “Event Transformer. A sparse-aware solution for efficient event data processing,” in IEEE/CVF Conference on Computer Vision and Pattern Recognition Workshops (New Orleans, LA: IEEE), 2676–2685. 10.1109/CVPRW56347.2022.00301

[B24] Szegedy C. Vanhucke V. Ioffe S. Shlens J. Wojna Z. (2016). “Rethinking the inception architecture for computer vision,” in 2016 IEEE Conference on Computer Vision and Pattern Recognition (Las Vegas, NV: IEEE Computer Society), 2818–2826. 10.1109/CVPR.2016.308

[B25] Wang Y. Zhang X. Shen Y. Du B. Zhao G. Cui L.. (2022). Event-stream representation for human gaits identification using deep neural networks. IEEE Trans. Pattern Anal. Mach. Intell. 44, 3436–3449. 10.1109/TPAMI.2021.3054886 33502972

[B26] Wu J. Chua Y. Zhang M. Li G. Li H. Tan K. C. (2021). A tandem learning rule for effective training and rapid inference of deep spiking neural networks. IEEE Trans. Neur. Netw. Learn. Syst. 34, 446–460. 10.1109/TNNLS.2021.3095724 34288879

[B27] Wu Y. Deng L. Li G. Zhu J. Shi L. (2018). Spatio-temporal backpropagation for training high-performance spiking neural networks. Front. Neurosci. 12, 331. 10.3389/fnins.2018.00331 29875621 PMC5974215

[B28] Wu Z. Zhang H. Lin Y. Li G. Wang M. Tang Y. (2021). LIAF-Net: Leaky integrate and analog fire network for lightweight and efficient spatiotemporal information processing. IEEE Trans. Neur. Netw. Learn. Syst. 33, 6249–6262. 10.1109/TNNLS.2021.3073016 33979292

[B29] Yao M. Gao H. Zhao G. Wang D. Lin Y. Yang Z. -X.. (2021). “Temporal-wise attention spiking neural networks for event streams classification,” in 2021 IEEE/CVF International Conference on Computer Vision (Montreal, QC: IEEE), 10201–10210. 10.1109/ICCV48922.2021.01006

[B30] Zhang W. Li P. (2019). “Spike-train level backpropagation for training deep recurrent spiking neural networks,” in Advances in Neural Information Processing Systems 32: Annual Conference on Neural Information Processing Systems 2019, eds H. M. Wallach, H. Larochelle, A. Beygelzimer, F. d'Alche-Buc, E. B. Fox, and R. Garnett (Vancouver, BC: NeurIPS), 7800–7811. Available online at: https://proceedings.neurips.cc/paper/2019/hash/f42a37d114a480b6b57b60ea9a14a9d2-Abstract.html

[B31] Zhang W. Li P. (2021). “Spiking neural networks with laterally-inhibited self-recurrent units,” in International Joint Conference on Neural Networks (Shenzhen: IEEE), 1–8. 10.1109/IJCNN52387.2021.9533726

[B32] Zheng H. Wu Y. Deng L. Hu Y. Li G. (2021). “Going deeper with directly-trained larger spiking neural networks,” in Thirty-Fifth AAAI Conference on Artificial Intelligence, AAAI2021, Thirty-Third Conference on Innovative Applications of Artificial Intelligence, IAAI 2021, The Eleventh Symposium on Educational Advances in Artificial Intelligence, EAAI 2021 (AAAI Press), 11062–11070.

[B33] Zhu L. Wang X. Chang Y. Li J. Huang T. Tian Y. (2022). “Event-based video reconstruction via potential-assisted spiking neural network,” in IEEE/CVF Conference on Computer Vision and Pattern Recognition (New Orleans, LA: IEEE), 3584–3594. 10.1109/CVPR52688.2022.00358

